# Effects of the Thyroid Endocrine System on Gonadal Sex Ratios and Sex-Related Gene Expression in the Pufferfish *Takifugu rubripes*

**DOI:** 10.3389/fendo.2021.674954

**Published:** 2021-05-07

**Authors:** Zhen Yuan, Xufang Shen, Hongwei Yan, Jieming Jiang, Binwei Liu, Lei Zhang, Yumeng Wu, Ying Liu, Qi Liu

**Affiliations:** ^1^ College of Fisheries and Life Science, Dalian Ocean University, Dalian, China; ^2^ Key Laboratory of Environment Controlled Aquaculture, Ministry of Education, Dalian, China; ^3^ College of Marine Science and Environment Engineering, Dalian Ocean University, Dalian, China; ^4^ College of Life Science, Liaoning Normal University, Dalian, China

**Keywords:** *Takifugu rubripes*, methimazole, thyroid hormone, sex differentiation, RNA-seq

## Abstract

To examine the effect and mechanism of thyroid hormone on gonadal sex differentiation, *Takifugu rubripes* larvae were treated with goitrogen (methimazole, MET, 1000 g/g), and thyroxine (T4, 2nM) from 25 to 80 days after hatching (dah). Gonadal histology and sex ratios of fish were then determined at 80 dah. MET treatment induced masculinization, but T4 treatment did not induce feminization in *T. rubripes* larvae. Transcriptomic analysis of gonads at 80 dah was then conducted. Among the large number of differentially expressed genes between the groups, the expression of *foxl2*, *cyp19a1a*, and *dmrt1* was altered. The expression of *foxl2*, *cyp19a1a*, *dmrt1* and *gsdf* at 25, 40, 55 days after treatment (dat) was further analyzed by qPCR. MET treatment suppressed the expression of *foxl2* and *cyp19a1a*, and induced the expression of *dmrt1* in genetic females (*p* < 0.05). Additionally, T4 treatment induced an increase in the expression of *cyp19a1a* in genetic XY gonads only at 25 dat. However, the increase in *cyp19a1a* expression did not continue to 40 and 55 dat. This may explain why feminization of larvae was not found in the T4-treated group. Thus, the present study provides the first evidence that MET treatment causes masculinization in teleost fish. The effects of MET-induced masculinization in *T. rubripes* may act primarily *via* suppression of the expression of *foxl2* and *cyp19a1a*, and stimulation of the expression of *dmrt1*. Moreover, the effects of higher concentrations of T4 or different concentrations of T3, on sex differentiation require further testing.

## Introduction

Sex determination and differentiation, which develops in either the ovary or testis, is one of the most fundamental and surprisingly diverse biological processes. Across vertebrates, sex differentiation exhibits great variability and diversity. In mammals, sex determination and differentiation are strictly genetically controlled where the sex of the offspring is decided at the moment of fertilization the differential inheritance of sex chromosomes. In contrast, sexual differentiation of teleost vertebrates often happens in response to external signals (eg. social cues, temperature, light, or pH) at an early stage of development or even in adulthood (1,4). Endocrine processes play a critical role in sex determination, and gonadal fate. For example, during the critical period of sex differentiation, sex steroids regulate of steroidogenic enzyme genes by certain transcription factors that are essential for gonadal fate and sex (5). In *Odontesthes bonariensis* and the *Paralichthys olivaceus*, glucocorticoids were shown to downregulate *cyp19a1a* expression resulting in testicular differentiation (6, 7). Additionally, in *P. olivaceus*, cortisol was involved in suppressing *cyp19a1a* expression by binding to glucocorticoid responsive elements upstream of the *cyp19a1a* promoter (7).

Thyroid hormones (THs) have been well demonstrated in regulating various biological processes, such as morphogenesis, growth, reproduction, osmoregulation, and skin pigmentation (8). However, the role of TH in fish gonadal sex differentiation has been explored in only two species of teleost, the three-spined stickleback (*Gasterosteus aculeatus*) (8) and zebrafish (*Danio rerio*) (9,11). In *G. aculeatus*, TH-synthesis inhibitor (perchlorate) treatment induced hermaphroditism in genetic females (12). Exposure of *D. rerio* larvae to thyroxine during the early stages of sex differentiation causes male-biased populations in a concentration-dependent manner. However, goitrogen treatment did not cause a permanent feminization, but results in some delays in the ovary-to-testis transformation of genetic males (9, 10). These results leave open the question of whether TH plays a consistent role in the regulation of sex differentiation in teleosts. Among amphibians, goitrogens treatment was shown to induce gonadal masculinization (13) or feminization (13, 14), depending on the species. Sharma etal. further examined the effects of TH on the expression of several sex-related genes involved in gonadal sex differentiation in *D. rerio* (11). The results showed that T4 induced masculinizing in larvae by inhibiting female genes such as *cyp19a1a*, *esr1*, *esr2a*, and *esr2b*, and stimulating male sex-related genes *amh* and *ar*. Meanwhile, goitrogen (methimazole, MET) treatment increased the expression of *esr1*, *esr2a*, and *esr2b*, and reduced the expression of *amh* and *ar*. However, the inability of inducing *cyp19a1a* expression may explain the reversible feminizing activity of goitrogen on gonadal sex determination. Knowledge of the roles of non-classical hormones such as TH during gonadal sex differentiation would be of value not only to the understanding of the process of sex determination and differentiation, but also to the advancement of comparative endocrinology and development. However, the molecular changes of the effects of thyroid endocrine manipulation have still not been widely investigated either in amphibian or teleosts.

The tiger pufferfish, *Takifugu rubripes*, is one of the most valuable commercial fish cultured in Asia. On the market, male fish are more highly valued than females since the mature testes of tiger pufferfish are regarded as a delicacy. Therefore, all-male stocks are preferred in aquaculture. *T. rubripes* is a gonochoristic fish having an XX/XY sex determination system. This species has the most compact genome sequence, and an allelic variation in the *amhr2* gene, which codes for the amh receptor, is responsible for maleness (15, 16). With this knowledge, *T. rubripes* is an excellent organism to investigate genetic function in relation to sex determination and differentiation. Similar to other teleosts, sex steroids hormones and temperature seem to modulate the process of gonadal sex differentiation in this species (17,20). However, the involvement of non-classical hormones such as TH on sex differentiation has not been examined in *T. rubripes*. Thus, this study aims to investigate the role of TH using MET as an inhibitor of thyroid function and exogenous treatment with T4. This study may provide a basis for gaining a comprehensive understanding of the molecular mechanisms underlying thytoxine (T4) or MET treatment. In this way, the regulatory network might be elucidated at the critical stage of molecular sex differentiation in *T. rubripes*.

## Materials and Methods

### Treatment of *T. rubripes* Larvae and Sampling

*T. rubripes* larvae at 20 days after hatching (dah) were obtained from a fishery in Dalian (Dalian Fugu Aquatic Product Co., Ltd) in March 2019. All animal experiments were performed according to the Guide for the Care and Use of Laboratory Animals in Dalian Ocean University, Dalian, China. All animal experiments were approved by the animal study ethical committee of Dalian Ocean University and they are in compliance with Chinese laws, regulations, and ethics. After accommodation for 5 days, *T. rubripes* were randomly (950 larvae/tank) divided into three groups, including the control group, the MET-treated group, and the TH-treated group. All conditions were done with three replicates. For the MET treatment, larvae were fed with a commercial diet mixed with MET (1000 g/g diet, Sigma-Aldrich, USA) from 25 to 80 dah. In the T4 treatment group, larvae were immersed in aquaria containing T4 (Sigma-Aldrich) with a final concentration of 2 nmol/mL from 25 to 80 dah. Briefly, 0.45ml stock solutions (400 nmol/mL) were added to each tank (with 90 L water) to give final immersion concentrations of 2 nmol/L. After immersion, the water was renewed twice (80 L/change) to remove residual hormone. The treatment concentration was based on previous studies in zebrafish (11). *T. rubripes* larvae were fed a commercial diet (SanTong Company, Weifang, China) with or without treatment six times a day, and were reared at 21-22C under a natural photoperiod. The bottom of the tank was cleaned to remove debris, excess feed, and dead larvae, and the water was renewed twice daily to maintain the quality of the rearing water. Salinity, temperature, dissolved oxygen, and pH were monitored daily. The larvae were maintained at a salinity of 33 ppt. The oxygen level was maintained > 8 mg L^1^ and the pH was maintained at 7.98.1. Ammonia and nitrites were measured in water samples taken from the tanks weekly, and the mean values were always < 0.2 and < 0.05 mg L^1^, respectively. The bottom of the tank was cleaned to remove debris, excess feed, and dead larvae, and the water was renewed twice daily to maintain the quality of the rearing water.

At 40 and 55 days after treatment (dat), ten larvae from each tank were chosen randomly to be anesthetized on ice. The body length and wet weight were measured. Based on the daily mortality and the number of sampling, final observed survival rate (%) estimates were calculated according to the previous study (21). For histological observations, tissue samples (trunks that contained the gonads) were collected at 55 dat (n = 30/treatment) and fixed in Bouins solution. Additionally, gonads of 100 individuals at 25, 40, and 55 dat were dissected and each gonad was placed into a tube with 100 l of RNAlater reagent (Thermo Fisher Scientific, Baltics, USA). Samples were stored at 80C prior to RNA extraction. In order to identify the sex of each individual, a piece of tissue sample was stored in a 1.5 tube containing 100% alcohol in the freezer at 20C.

### Histology of Gonads, Genetical Sex Identification, and RNA Extraction

After fixation in Bouins fluid for 24h, gonads were transferred to 70% ethanol, and then samples were dehydrated in ascending concentrations of ethanol. Dehydrated samples were embedded in paraffin. Tissue sections of 6-m thickness were cut and stained with hematoxylineosin. Photographs were taken using a Nikon ECLIPSE Ci-L microscope (Tokyo, Japan). To distinguish the sex-reversal fish, the remained paraffin blocked tissue from 55 dat after histological observation were subjected to DNA extraction using the TIANamp FFPE DNA kit (Tiangen, China). The genetic sex of each fish was identified using SNP markers (a region containing exon 9 of the *amhr2* gene) as described previously (15, 22). For transcriptomic analysis and qPCR experiments, genetic sex identification was performed before RNA extraction. DNA of each sample was extracted using the TIANamp Marine Animals DNA kit (Tiangen).

For RNA extraction, ten ovaries or ten testes were pooled for RNA extraction in each sampling point using the Qiagen RNeasy Micro Kit (Qiagen, USA). After genomic DNA elimination (DNAfree kit, Qiagen), RNase inhibitor (Takara, Japan) was added into the purified RNA samples before they were stored at 80C. RNA purity and integrity were examined with a NanoDrop ND-1000 spectrophotometer (Thermo Scientifc, Wilmington, DE, USA) and an Agilent 2100 Bioanalyzer (Agilent Technologies, Santa Clara, CA, USA).

### RNA-Sequencing

For each RNA sample preparation, 1 g RNA of *T. rubripes* gonads at 55 dat was used as input material. In total, six sequencing libraries were generated using the NEBNext Ultra RNA Library Prep Kit for Illumina (NEB, USA) following the manufacturers recommendations as described elsewhere (22). The libraries included control genetic female (C_XX), control genetic male (C_XY), MET-treated female (MET_XX), MET-treated male (MET_XY), T4-treated female (T4_XX) and T4-treated male (T4_XY). The library fragments were purified with the AMPure XP system (Beckman Coulter, Beverly, USA) to select cDNA fragments of preferentially 250-300 bp in length. Subsequently, size-selected, adaptor-ligated cDNA were mixed with 3 l USER Enzyme (NEB) for 15min at 37C followed by 5min at 95C before PCR. Then, Phusion High-Fidelity DNA polymerase, Universal PCR primers and Index (X) Primer were used for PCR. Finally, after purifying PCR products using the AMPure XP system, the library quality was evaluated using an Agilent Bioanalyzer 2100 system. Index codes were added to attribute sequences to each sample. The clustering of the index-coded samples was carried out on a cBot Cluster Generation System according to the manufacturers instructions (TruSeq PE Cluster Kit v3-cBot-HS, Illumia). After cluster generation, all six libraries were sequenced on an Illumina Novaseq platform and 150 bp paired-end reads were generated. The reference genomes were downloaded from the website ftp://ftp.ncbi.nlm.nih.gov/genomes/all/GCF_000180615.1_FUGU5/. Clean data from each library were aligned to the genome of *T. rubripes* using Hisat2 v2.0.5. Fragments per kilobase of exon models per million mapped reads, based on the length of the gene and read count mapped to this gene, were used for calculating gene expression levels. DESeq2 R package was used for differential expression analysis of two libraries. The resulting P values were adjusted using the Benjamini, Hochberg method (23). Corrected *P*-value of 0.05 and absolute fold change of 2 was set as the threshold for significantly differential expression. Differentially expressed genes (DEGs) were categorized based on Gene Ontology (GO) and Kyoto Encyclopedia of Genes and Genomes (KEGG) enrichment analysis. GO terms with corrected *P* value less than 0.05 were considered significantly enriched by differential expressed genes.

### qPCR Verified

Primers for qPCR were designed using Primer Premier 5.0 program (**Table 1**). The relative abundance of the selected gene was evaluated based on the 2^CT^ method. The levels of *foxl2*, *cyp19a1a*, *dmrt1*, and *gsdf* (a gene identified in our previous study) in the gonads of larvae at 25, 40, and 55 dat were determined in each group using the Applied Biosystems 7900 HT Real-Time PCR System. The stability of two commonly used reference genes *-actin* and *elongation factor 1-alpha* (*ef1*) of *T. rubripes* was evaluated using Normfinder (v 0.953) (17,20). The evaluation revealed that *ef1* is more stable reference gene in this study. Hence, *ef1* was used as a reference gene in the qPCR analysis as described previously (22).

**Table 1:** Primers used for qPCR of *-actin* and sex-related genes of *Takifugu rubripes*.

Name	Primer	Sequence (53)	Length (bp)
*ef1*	Forward	AGGAGGGCAATGCTAGTGG	204
	Reverse	TGGTCAGGTTGACGGGAG	
*foxl2*	Forward	GTATCAGGCACAACCTGAGTCTC	125
	Reverse	GTTGCCCTTCTCAAACATATCCT	
*cyp19a1*	Forward	ATTCACCAGAAGCACAAGACG	118
	Reverse	CAGTGAAGTTGATGTTCTCCAGT	
*dmrt1*	Forward	ATGGTTACCTCCGATCTGCAC	125
	Reverse	AACTTGGAGTTCCTTCCCATG	
*gsdf*	Forward	TCTTATGTCTGCTGTGTTTCCTC	147
	Reverse	TTACAGGGCTCTTGTAATTTGTG	

### Statistical Analyses

One-way ANOVA followed by the Duncans test (IBM SPSS statistics version 22.0, IBM, Chicago, IL, USA) was performed to examine the statistical significance of growth, survival rate and gene expression levels between control groups and treatment groups, respectively. A *p* value of < 0.05 was considered significant.

## Results

### Growth, Survival Rate of the Larvae, and Histological Analysis of the Gonads

**Figure 1:**
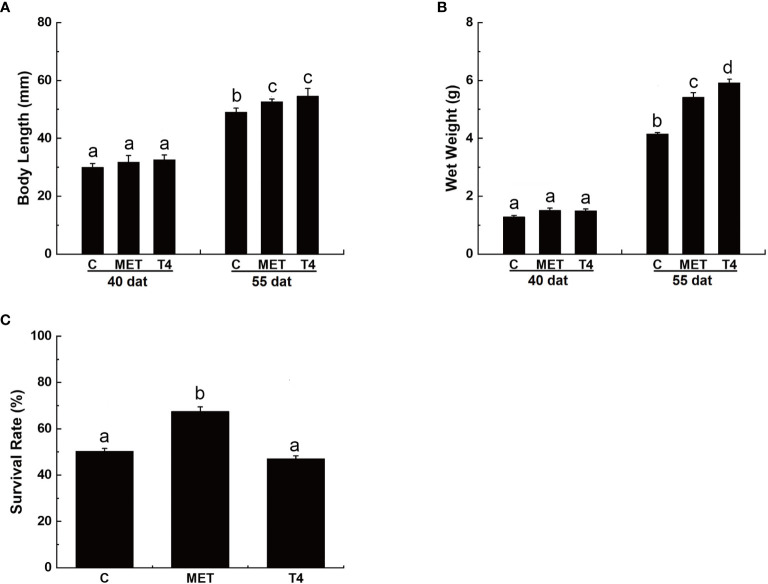
Growth and survival rate (%) of *Takifugu rubripes* larvae in different experimental groups. **(A)** Body length of *T. rubripes* larvae at 40 and 55 days after treatment (dat). **(B)** Wet weight of *T. rubripes* larvae at 40 and 55 dat. **(C)** Survival rate of *T. rubripes* larvae at 55 dat. C, control; MET, MET-treated group; T4, T4-treated group. Each value represents the mean SD of three measurements, different lowercase letters indicate significant differences between each treatment (one-way ANOVA, *P* < 0.05, n = 3).

At 40 dat, the body length of larvae in the T4 p (32.63 0.61mm) and MET treatment groups (31.84 2.60mm) was similar to the control group (29.98 1.33mm) (*p* > 0.05) (**Figure 1A**). No significant difference in wet weight was observed between the treatment and control groups (*p* > 0.05) (**Figure 1B**). At 55 dat, the body length of larvae in the T4 (54.57 2.66mm) and the MET (52.62 1.26mm) treatment groups was significantly higher than the control group (49.06 1.33mm) (*p* < 0.05) (**Figure 1A**). The wet weight of larvae in the T4 (5.92 0.12g) and the MET (5.42 0.16g) treatment group were significantly higher than the control group (4.15 0.05g) (*p* < 0.05) at 55 dat (**Figure 1B**).

By 55 dat, no significant difference in the survival rate of larvae was observed in the T4-treated group compared with the control group (*p >* 0.05). However, the survival rate of larvae in the MET treatment group was significantly higher(67.54% 1.93%) than the control group (50.33% 1.14%) (*p* < 0.05) (**Figure 1C**).

**Figure 2:**
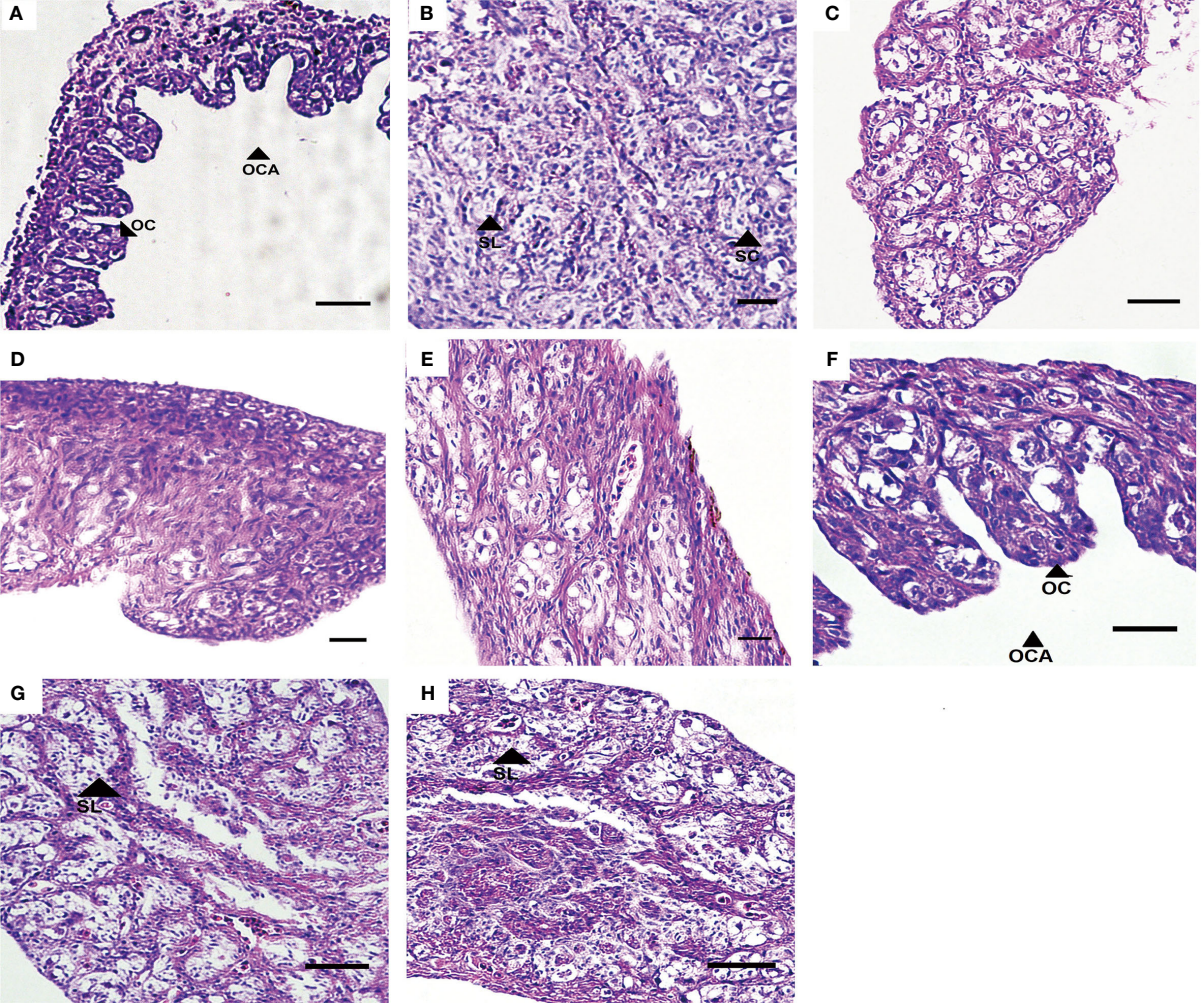
Gonad development in *T. rubripes* at 55 dat. **(A)** and **(B)**, XX and XY of control groups; **(C)** and **(D)**, MET-treated XX, **(E)**, MET-treated XY; **(F)**, T4-treated XX; **(G)** and **(H)**, T4-treated XX. OC, oocyte, OCA, ovarian cavity, SL, spermatogenic cysts, SC, spermatocyte. Scale bar, 20 m.

At 80 dah (55 dat) no sex reversal phenomenon was observed in the control group, while the oviposition plate and the ovary cavity were clearly observed in the ovary (**Figure 2A** and **Table 2**). There was a large amount of oogonia and a small number of oocytes on the oviposition plate. In the testis, along the circumference of the testes, a large number of spermatocytes was observed, but no sex reversal larvae was found (**Figure 2B** and **Table 2**). MET successfully induced 100% masculinization, and all the samples had intermediate gonads having empty lumina (**Figures 2CE** and **Table 2**). In the T4-treated group, no sex reversal larvae were found (**Figures 2FH** and **Table 2**).

**Table 2:** Sex ratio of *T. rubripes* at 55 dat in different treatment groups according to the phenotype of gonads.

Group	Male	Female	Intermediate
Control	12	18	0
MET-treated group	0	0	30
T4-treated group	14	16	0

### Sequencing and Mapping

Raw reads of 45,874,794 (C_XX), 45,793,670 (C_XY), 47,301,088 (MET_XX), 47,429,562 (MET_XY), 46,075,656 (T4_XX), and 47,710,900 (T4_XY) were generated by sequencing the six cDNA libraries derived from gonads of *T. rubripes* from three experiment groups, respectively (**Table 3**). The raw sequence reads have been submitted to the Short Read Archive with the following accession numbers: SRR12364833, SRR12358231, SRR12364848, SRR12364847, SRR12364850, and SRR12364849. After filtering out low-quality data, clean reads (44, 634, 680 in C_XX, 44, 951, 110 in C_XY, 46, 011, 968 in MET_XX, 46, 634, 316 in MET_XY, 44, 872, 284 in T4_XX, and 46, 537, 592 in T4_XY) were obtained. The data showed that 90.87%, 90.42%, 90.79%, 88.30%, 90.65%, and 90.75% of the clean reads were uniquely mapped to the FUGU genome (**Table 3**).

**Table 3:** Summary statistics of the transcriptome sequencing and mapping in *T. rubripes*.

Sample	Raw data	Clean_reads	Total_map	Unique_map	Multi_map
C_XX	45,874,794	44,634,680	42,171,942(94.48%)	40,558,161(90.87%)	1,613,781(3.62%)
C_XY	45,793,670	44,951,110	42,239,017(93.97%)	40,644,819(90.42%)	1,594,198(3.55%)
MET_XX	47,301,088	46,011,968	43,445,083(94.42%)	41,774,672(90.79%)	1,670,411(3.63%)
MET_XY	47,429,562	46,634,316	44,060,019(94.48%)	41,179,089(88.3%)	2,880,930(6.18%)
T4_XX	46,075,656	44,872,284	42,317,717(94.31%)	40,674,741(90.65%)	1,642,976(3.66%)
T4_XY	47,710,900	46,537,592	43,913,927(94.36%)	42,231,369(90.75%)	1,682,558(3.62%)

### Global Gene Expression Profiles in the Different Groups

**Figure 3:**
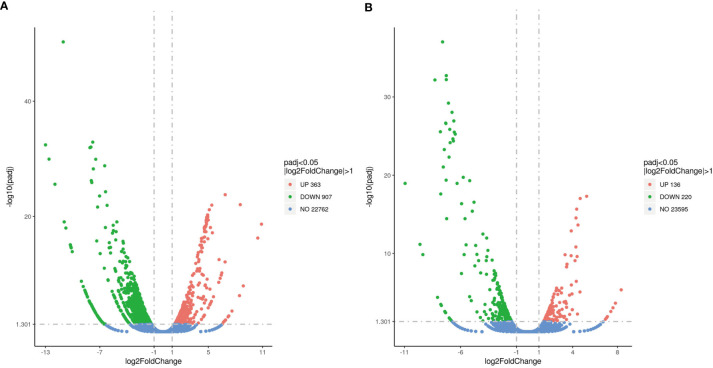
Volcano of DEGs for MET_XX versus C_XX **(A)**, and T4_XY versus C_XY **(B)**.

**Figure 4:**
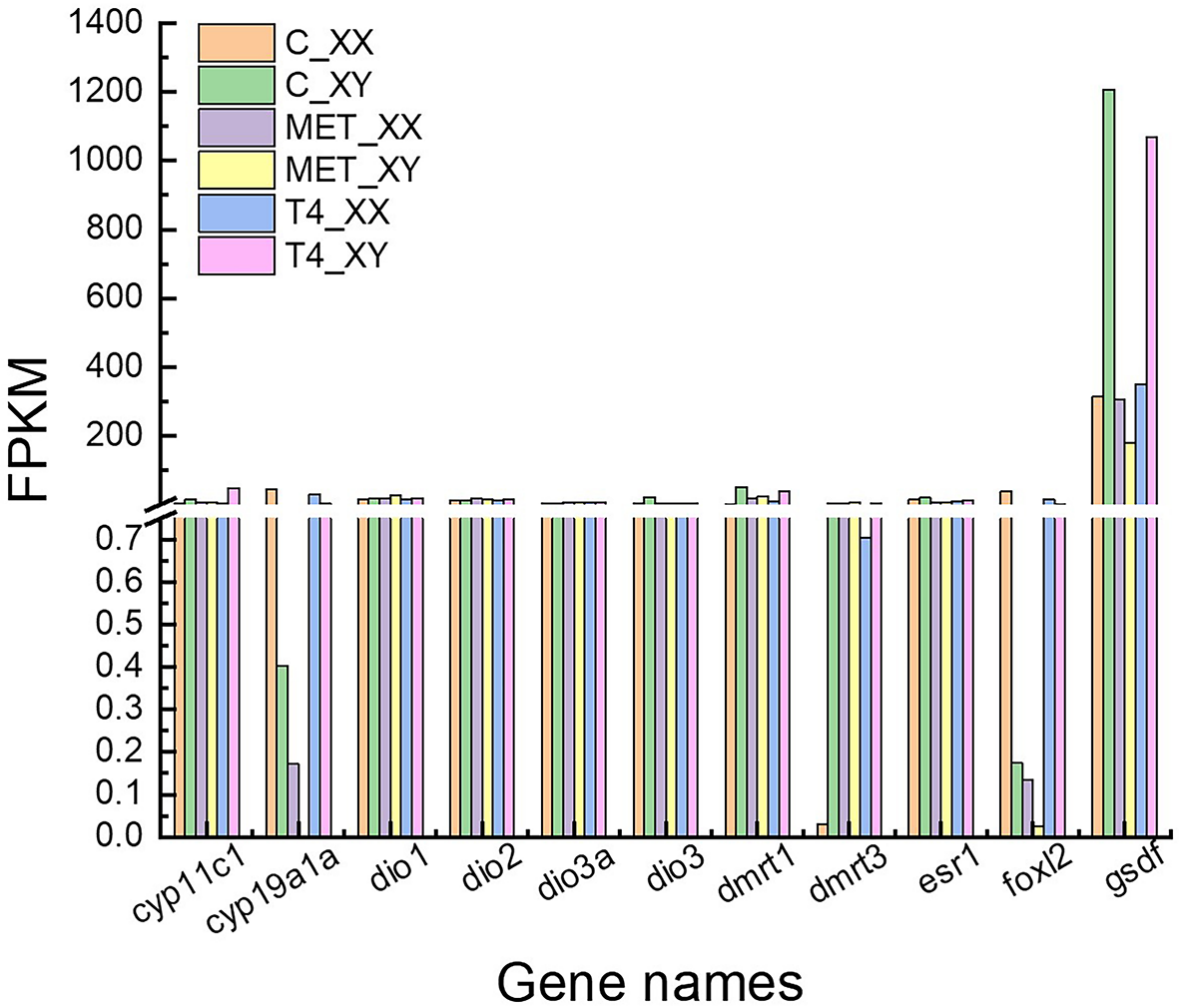
FPKM (reads per kb per million reads) of *cyp19a1a*, *dio1*, *dio2*, *dio3a*, *dmrt1*, *dmrt3*, *esr1*, *foxl2*, *gsdf*, *thr*, *thr* and *cyp19a1a* obtained by RNA-seq.

Approximately 1,270 DEGs were identified between the MET_XX and C_XX, including 907 down-regulated genes, including *cyp19a1*, *zp3-like*, *zp4-like*, *foxl2*, *hsd17b1*, *P450scc*, *piwi2, pgr, cyp17a2*, and *esr1*. Likewise, 363 up-regulated genes, such as *dmrt1*, *dmrt3*, and *lhcgr* were identified (**Figures 3A**, **4**, and **Table 4**). Between T4_XY and C_XY, 356 DEGs were identified, of which 220 genes were down-regulated, such as *cyp17a1*, and *cyp11c1*, and 136 genes were up-regulated, such as *hsp70*, *cyp19a1a*, *foxl2*, and *hsp90a* (**Figures 3B**, **4**, and **Table 4**). Moreover, no sexual dimorphic expression of three deiodinases (*dio1*, *dio2*, *dio3a*), and two thyroid receptors (*tr* and *tr*) have been found between testes and ovaries, and the changes in their expression were not found after T4 or MET treatment by RNA-Seq. Yet, dio3 was identified as DEGs between C_XX and C_XY fugu, and both T4 and MET treatment downregulated its expression in XY *T. rubripes*.

**Table 4:** Representative DEGs in MET_XX vs C_XX and T4_XY vs C_XY.

Genes name	log2 Fold change	Description	
****	**MET_XX vs C_XX**	
*cyp11c1*	-2.83	cytochrome P450 11B%2C mitochondrial	
*cyp17a2*	-2.74	cytochrome P450 family 17 polypeptide 2	
*dmrt1*	3.72	doublesex and mab-1 related transcription factor 1	
*dmrt3*	6.49	doublesex and mab-3 related transcription factor 3	
*esr1*	-1.42	estrogen receptor 1%2C transcript variant X2	
*foxl2*	-7.24	forkhead box L2
*hmgcll1*	-2.32	3-hydroxymethyl-3-methylglutaryl-CoA lyase-like 1	
*hsd17b1*	-5.19	hydroxysteroid (17-beta) dehydrogenase 1	
*igfbp1*	-3.19	insulin-like growth factor binding protein 1	
*lhcgr*	1.71	luteinizing hormone/choriogonadotropin receptor	
*mGluR6*	-2.91	metabotropic glutamate receptor 6-like	
*cyp19a1a*	-7.98	cytochrome P450 19A1-like%2C transcript variant X1	
*per3*	-1.53	period circadian clock 3%2C transcript variant X1	
*P450scc*	-3.43	cholesterol side-chain cleavage enzyme	
*piwi2*	-2.92	piwi-like RNA-mediated gene silencing 2	
*pgr*	-1.75	progesterone receptor	
*th*	1.46	tyrosine hydroxylase	
*tshr*	-2.49	thyroid stimulating hormone receptor	
*ssr2*	-2.06	somatostatin receptor type 2	
*zp3-like*	-12.62	zona pellucida sperm-binding protein 3-like	
*zp4-like*	-11.96	zona pellucida sperm-binding protein 4-like	
	**T4_XY vs C_XY**
*cyp11c1*	-1.77	cytochrome P450 11B%2C mitochondrial	
*cyp17a1*	-1.93	cytochrome P450 family 17 polypeptide 1	
*foxl2*	2.35	forkhead box protein L2	
*hsp70*	4.67	heat shock 70 kDa protein 1	
*hsp90a*	2.17	heat shock protein HSP 90-alpha	
*cyp19a1a*	3.01	cytochrome P450 19A1-like%2C transcript variant X1	

**Figure 5:**
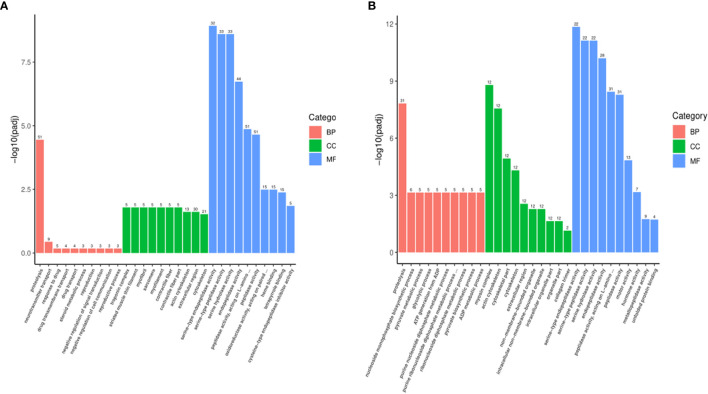
Gene ontology (GO) classifications of DEGs for MET_XX versus C_XX **(A)**, and T4_XY versus C_XY **(B)**.

The most significantly enriched top 30 terms are shown in **Figure 5**. In MET_XX versus C_XX (**Figure 5A**), and T4_XY versus C_XY (**Figure 5B**), the number of GO terms in the molecular function was most prevalent, followed by biological process and cellular component. In MET_XX versus C_XX, genes involved in peptidase activity, acting on L-amino acid peptides, peptidase activity, and endopeptidase activity were highly represented for the molecular function; extracellular region and cytoskeleton were highly represented for the cellular component; proteolysis was highly represented for the biological process. In T4_XY versus C_XY, peptidase activity, acting on L-amino acid peptides, peptidase activity, and endopeptidase activity were highly represented for the molecular function; myosin complex, actin cytoskeleton, extracellular region, non-membrane-bounded organelle, etc. were highly represented for the cellular component; proteolysis was highly represented for the biological process.

**Figure 6:**
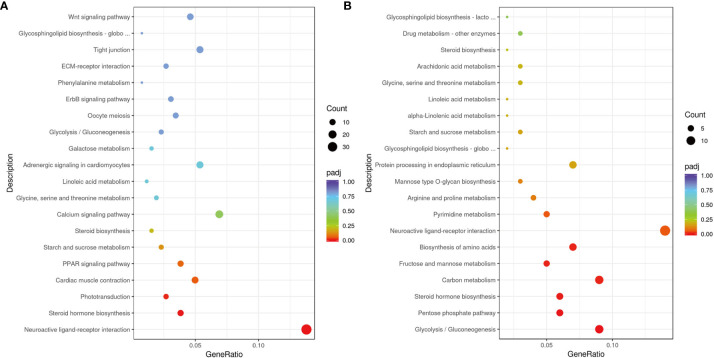
KEGG enrichment analyses of DEGs for MET_XX versus C_XX **(A)**, and T4_XY versus C_XY **(B)**.

According to KEGG analysis, the top 20 most enriched KEGG signaling pathways between groups are shown in **Figure 6**. In MET_XX versus C_XX, the most enriched KEGG pathways were neuroactive ligand-receptor interaction, calcium signaling pathway, cardiac muscle contraction, adrenergic signaling in cardiomyocytes, wnt signaling pathway, tight junction, steroid hormone biosynthesis, and phototransduction. In T4_XY versus C_XY, the most enriched KEGG pathways were neuroactive ligand-receptor interaction, glycolysis/gluconeogenesis, carbon metabolism, biosynthesis of amino acids, steroid hormone biosynthesis, protein processing in endoplasmic reticulum.

### The Expression Profiles of *foxl2*, *cyp19a1a*, *dmrt1*, and *gsdf* in Response to MET and T4 Treatment by qPCR

**Figure 7:**
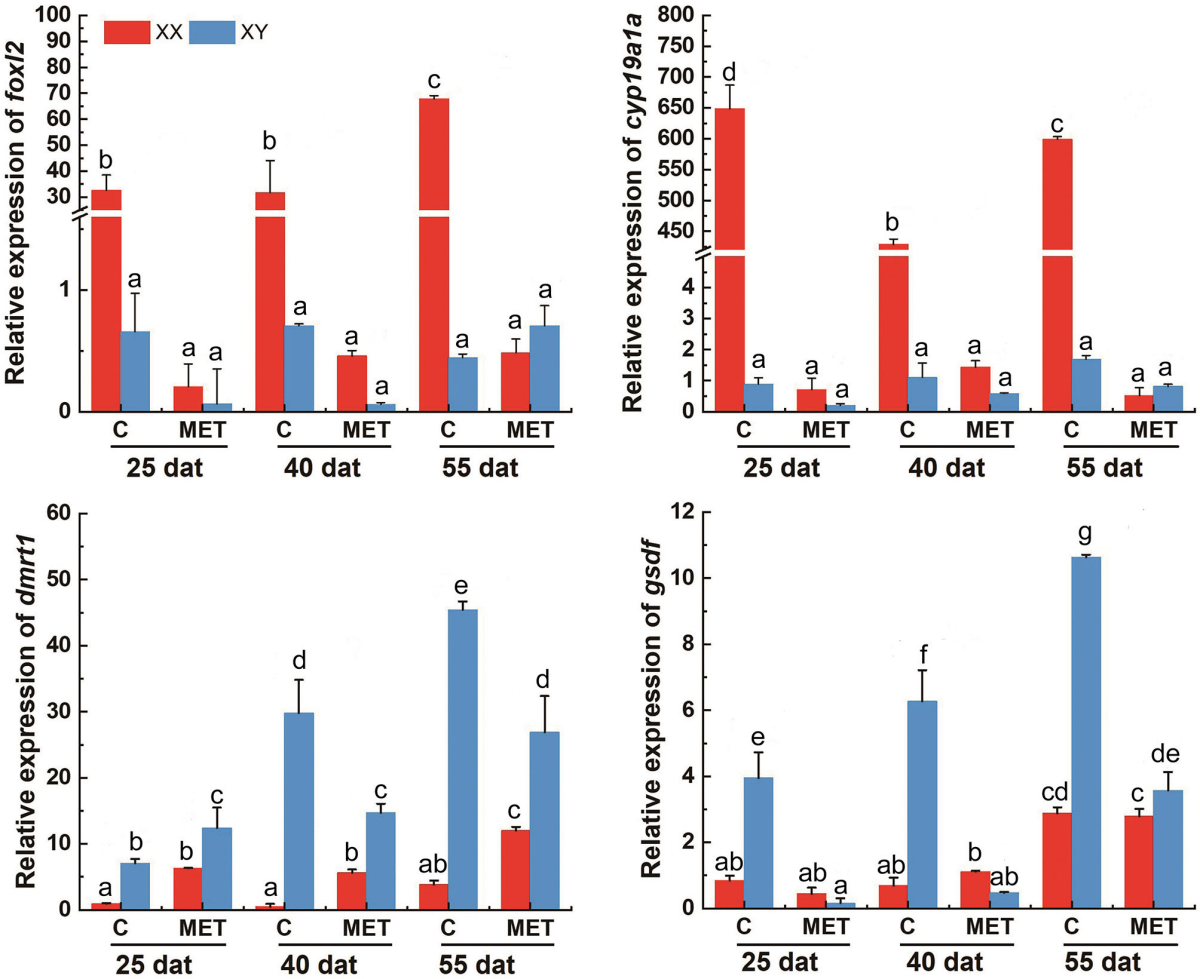
The levels of *foxl2*, *cyp19a1a*, *dmrt1*, and *gsdf* expression in *T. rubripes* gonads after MET treatment. C, Control group; MET, MET-treated group. Each value represents the mean SD of three measurements, different lowercase letters indicate significant differences between each treatment (one-way ANOVA, *P* < 0.05, n = 3); dat, days after treatment.

**Figure 8:**
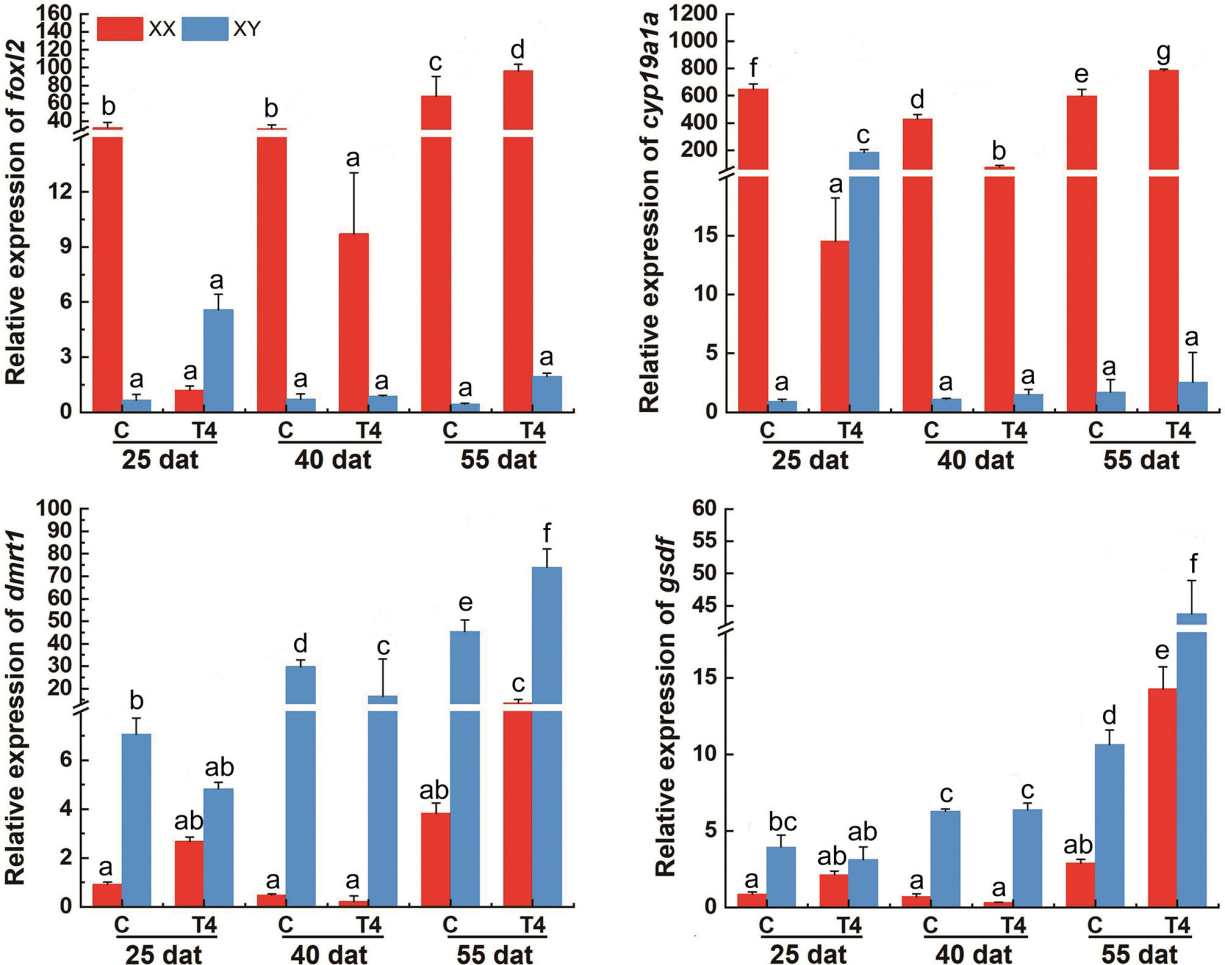
The levels of *foxl2*, *cyp19a1a*, *dmrt1*, and *gsdf* in *T. rubripes* gonads after T4 treatment. C, Control group; T4, T4-treated group. Each value represents the mean SD of three measurements, different lowercase letters indicate significant differences between each treatment (one-way ANOVA, *P* < 0.05, n = 3); dat, days after treatment.

In the control group, the expression levels of *foxl2* and *cyp19a1a* were significantly higher in XX gonads than that in XY gonads, while the expression level of *dmrt1* and *gsdf* mRNA were significantly higher in XY gonads than in XX gonads at 25, 40 and 55 dat (*p* < 0.05) (**Figures 7** and **8**). In the MET treatment group, the level of *foxl2* and *cyp19a1a* expression in the genetic XX gonads was immediately decreased from 25 dat. At 55 dat, the level of *foxl2* and *cyp19a1a* expression in MET treated-XX gonads was not significantly different from the control XY (*p* > 0.05). The expression of *dmrt1* increased in the gonads of MET-treated XX *T. rubripes* from 25 dat, but was significantly lower than that in genetic XY individuals in the control group and in the MET-treated group at 40, and 55 dat (*p* < 0.05). Compared with the control groups, the expression of *gsdf* in genetic XX individuals in the MET-treated group did not increase at 25, 40, and 55 dat (*p* > 0.05) (**Figure 7**). After T4 treatment, the expression levels of *cyp19a1a* genes in genetic XY were significantly increased compared with the genetic XY in the control group at 25 dat (*p* < 0.05). However, the increasing tendency was not continued at 40 and 55 dat. At 25, 40, and 55 dat, the level of *foxl2* expression in T4 treated-XY gonads was not significantly different from the control XY (*p* > 0.05). At 40 dat and 55 dat, the level of *cyp19a1a* expression in T4 treated-XY gonads was not significantly different from the control XY (*p* > 0.05). Moreover, compared to the control-XY, the significant decrease in the level of *dmrt1* expression in the T4-treated-XY was only observed at 40 dat (*p* < 0.05). The significant increase in the level of *gsdf* expression in T4-treated-XY was observed at 55 dat when compared with the control-XY (*p* < 0.05) (**Figure 8**). Interestingly, a decreased expression of *foxl2* and *cyp19a1a* in the T4-treated-XX was observed at 25 and 40 dat (*p* < 0.05).

## Discussion

By the end of the study, exposure to MET did not affect *T. rubripes* body length, but did increase body weight and survival rate compared with controls. However, in zebrafish, MET treatment was shown to reduce the body length of the larvae (24). In addition, exposure of fathead minnow eggs to 100 ppm ammonium perchlorate from 0 to 28 days post-fertilization caused a reduction in body length (25). Ammonium perchlorate inhibits TH synthesis by inhibiting iodide uptake into the thyroid follicles. However, the survival rate of fathead minnow and zebrafish was not affected by perchlorate treatment (9, 25). Moreover, in the present study T4 did not affect *T. rubripes* survival, but increased growth compared with controls. This finding is different from reports in zebrafish, where lethally toxic effects of T4 was observed. In this case, T4 treatment causes several developmental effects, such as an abnormal acceleration in lower jaw growth (9). Taken together, these results demonstrated the effects of TH and TH inhibitors on growth and survival is species-specific. Yet, it is unknown why T4 exposure induced higher larval mortality (9). Aggressive behavior or cannibalism that is commonly found in fish can lead to mass mortality of larvae (26, 27). Loss of fish can also be attributed to increased bacterial or fungal infections, or even by fish cannibalism (28,30). Larvae of *T. rubripes* exhibit aggressive behavior and cannibalism from 6 dah (31). In the rearing period, most of the dead fish showed signs of aggression including dermal bite marks. It is possible that MET may decrease potential cannibalism, which resulted in a lower overall mortality rate in our present study. However, how the level of TH effects on cannibalism needs further study in the near future.

Previous works on the effect of the thyroid on sex differentiation based on zebrafish and three-spined sticklebacks reported contradictory results. For example, treatment of zebrafish T4-induced hyperthyroid conditions produced more female offspring, while treatment with goitrogen, perchlorate or MET induced hypothyroid conditions result in more male offspring during early development (9,11). However, perchlorate-induced hypothyroid conditions caused functional hermaphrodites by masculinizing genetically female threespine sticklebacks (12). Similar controversial results of hypothyroid induction of female or male biased populations at the time of gonadal sex differentiation were also observed in amphibians (13, 14). The present study found that MET could induce masculinization. This finding provides the first evidence that MET treatment can cause masculinization in teleosts. This finding is contrary to previous studies in zebrafish (10). Moreover, although this study used a TH dosage that has been used in zebrafish, TH-induced sex reversal was not observed under the overall experimental conditions. Further study should be conducted using a higher dosage to clarify this issue. It is critical to understand the effects of TH on sex differentiation and its underlying molecular mechanism in more teleost species. Collecting the tiny gonads from larvae and juvenile fish is challenging and the total RNA that can be extracted from a single gonad in *T. rubripes* is often insufficient. Additionally, RNA sample pooling strategies have also been used in other teleosts such as Nile tilapia (32, 33). Pooling RNA samples is a good option for insufficient RNA and is effective in maintaining statistical power when testing DEGs with low-to-medium abundance levels (34). Therefore, pooled RNA samples were used for RNA-seq without biological repeats. Moreover, sex-related gene expression was examined by qPCR with pooled samples in triplicate. These genes exhibited similar expression profiles using both qPCR analysis and FPKM values by transcriptome analysis in treated and control fugu gonads, suggesting that we obtained high quality and convincing gonadal transcriptome data.

Larvae treated with MET showed a suppression of genes involved in steroid hormone biosynthesis, such as *foxl2* and *cyp19a1a* in genetic XX gonads. The expression of *foxl2* and *cyp19a1a* at 25, 40, and 55 dat was also compared between the MET-treated group and the control group using qPCR. The results were in accordance with the results of RNA-Seq. Cytochrome P450 aromatase, which is encoded by *cyp19a1a*, is responsible for the transformation of androgens into estrogens (predominantly E_2_) in the ovary (35,37). Blockage of endogenous estrogen synthesis *via* an aromatase inhibitor (e.g., fadrozole, letrozole) during the critical period of sex differentiation has been demonstrated to induce masculinization in a wide range of teleosts (38,42). Recent studies also indicated that long-term aromatase inhibitor treatment could induce a complete sex reversal in adult females of medaka (*Oryzias latipes*), tilapia (*Oreochromis niloticus*), and zebrafish (*D. rerio*) (43,45). This study reports that *cyp19a1a* exhibited a significantly higher level in female compared with male *T. rubripes* larvae (22). This is in accordance with previous studies. Treated with fadrozole from 19 to 100 dah also inhibited the ovarian cavity formation of all individuals, and induced the subsequently testicular differentiation in *T. rubripes* larvae (16). These results suggested that *cyp19a1a* may play a critical role in ovarian differentiation in *T. rubripes* as in other teleosts (38,45). Similarly, MET-induced masculinization may primarily occur through suppression of *cyp19a1a* and the production of E_2_. In zebrafish, hyperthyroid conditions that reduced male-biased sex ratios were accompanied by decreased expression of *cyp191a* and aromatase activity thereby generating male-biased gonadal sex ratios (11). In some mammals, hypothyroidism enhances aromatase activity and 17b-estradiol content in Sertoli cells of male rats (46, 47). Treatment with TH inhibits aromatase activity and estrogen production by the ovary (48, 49) and testicular Sertoli cells (46, 47). Therefore, these results indicate that the underlying mechanisms by which TH affects sex determination in *T. rubripes* and other teleosts may be related by intricate crosstalk between TH and the sex steroid hormones that appear to play a critical role in sex determination and differentiation in teleosts (50,53). In zebrafish, although MET delays testicular differentiation (ovary-to-testis transformation) in genetic males, and thyroid hormone induced masculinization of genetic females, both T4 and MET inhibited *cyp19a1a* expression (11). Such controversial results may point to an underlying mechanism of TH and MET on *cyp19a1a* expression and even sex differentiation. This effect may also be species-specific. One possible suggestion is that T4 may bind to the promoter of *cyp19a1a* to regulate its expression. Indeed, in silico analyses of the *T. rubripes cyp19a1a* promoter showed that there were three thyroid response elements bearing a palindromic sequence similar to what was identified as the thyroid binding site (5-TGACCT-3, 5-TGTCCT-3) in clawed frogs (*Silurana tropicalis*) (54).

*Foxl2* has been demonstrated function in the transcriptional regulation of *cyp19a1a* in Nile tilapia. Knockout of *foxl2* in XX fish by TALEN led to decreased *cyp19a1a* expression and serum E2 levels, resulting in sex reversal (55,57). Here, a higher expression of *foxl2* in MET-treated XX *T. rubripes* was observed compared with control XX. This may explain the increase of *cyp19a1a* expression. Moreover, one thyroid response element (5-TGTCCT-3) identified in *S. tropicalis*, was found in the promoter of *T. rubripes foxl2* by in silico analysis (54). Further experimental confirmation of putative thyroid response element in the promoter regions of *foxl2* and *cyp19a1a* in *T. rubripes* is required. Additionally, it was also found that T4 treatment induced an increased expression of *cyp19a1a* in genetic XY gonads at 25 dat. This result suggests that T4 was able to induce the expression of *cyp19a1a*. Conversely, the increased expression of *cyp19a1a* did not continue to 40 and 55 dat. This may explain why sex-reversal (testis to ovary) in larvae was not obtained in the T4-treated group. These results reinforced that further study should be conducted in future experiments. Additionally, the transcriptional analysis revealed that although the expression levels of *foxl2* and *cyp19a1a* of the T4-treated genetic XY were significantly lower than the genetic XX, levels were higher than genetic XY in the control group at 55 dat. This result differed from the results of qPCR experiment. This may be attributed to the tiny gonads of *T. rubripes* larvae that collected at 80 dah. This was due to the fact that different samples were used in the RNA-Seq and qPCR experiment.

In this study, treatment with MET increased the *dmrt1* expression in genetic XX *T. rubripes*. In silico analyses of *T. rubripes dmrt1* promoter showed that there were five thyroid response elements, which was identified as the thyroid binding site in clawed frogs (*Silurana tropicalis*) (54). In vertebrates, several DM domain genes (*dmrt* genes) have been shown to be required for gametogenesis and gonadal differentiation. *Dmrt1* seems to have a more significant role and is likely involved in testicular differentiation in all vertebrates (58). Our previous study reported that XY *T. rubripes* showed higher expression levels of *dmrt1* in undifferentiated gonads compared to XX individuals (22). During the early life stages of *T. rubripes*, 17-beta estradiol treatment induced a decrease of *dmrt1* and feminization in XY individuals (18, 19). Therefore, *dmrt1* may play a pivotal role during *T. rubripes* testicular differentiation. In addition to the negative regulation of *foxl2* and *cyp19a1a*, induction of male-biased sex ratios by MET may require stimulation of *dmrt1*. *dmrt1* has been shown to repress *cyp19a1a* transcription directly in tilapia. Gain-function analysis indicated that *dmrt1* overexpression in XX tilapia was able to decrease *cyp19a1a* expression and serum E_2_ levels that resulted in sex reversal (55). In medaka, a duplicate of the autosomal gene *dmrt1* encoding a DM-domain-containing transcriptional factor, *dmy*, may also be able to inhibit Ad4BP/SF-1 activated *cyp19a1a* gene transcription (55). Knockdown of *dmy* can also induce an increase of *cyp19a1a* expression and sex reversal (59). However, the relationship between *dmrt1* and *cyp19a1a* needs to be confirmed by future studies.

Results of the transcriptomic analysis showed that MET treatment can downregulate the expression of *estrogen receptor 1*. Estrogenics exert their function by binding to a specific receptor, the nuclear estrogen receptors (ERs) (60, 61). However, single knockout of *esr1*, *esr2a*, or *esr2b* did not result in sex reversal in zebrafish. While, double knockout of *esr2a/b* induced an arrest of folliculogenesis at the previtellogenic stage and subsequently affected the female-to-male sex reversal (62). Although *cyp19a1a* and both *esr2a/b* mutants induced female-to-male sex reversal, lack of aromatase inhibited ovarian differentiation whereas the loss of ERs failed to maintain ovarian status resulting in sex reversal. Their mechanisms were suggested to be different. Taken together, *cyp19a1a* is a reliable early marker of ovarian differentiation and is indispensable for ovarian differentiation/maintenance in fish. Thus, it was uncertain whether the down-regulation of *er1* was a cause or a consequence of MET-induced masculinization. *gsdf*, a new TGFB superfamily member found only in fish, has been cloned in many gonochoristic (63,66) and hermaphroditic fish (67,69). In *Oryzias luzonensis*, *gsdf* on the Y chromosome has been confirmed to be a sex-determining gene (65). Gain- and loss- functional analysis demonstrated that this gene essential for teleost sex determination and differentiation (70,72). In the male undifferentiated gonads of *T. rubripes*, higher expression levels of *gsdf* were observed, suggesting that it may have a similar role as described in other teleosts (22). In silico analyses of *T. rubripes gsdf* promoter showed that there was one thyroid response element (5-TGTCCT-3) was identified as the thyroid binding site (46). Yet, *gsdf* expression is not sensitive to either MET or TH exposure. This suggests that changes in *gsdf* expression are not necessary for, nor associated with, TH-dependent masculinization in *T. rubripes*.

Deiodinases are responsible for TH peripheral metabolism. Thyroid receptors mediate TH activity at sites of action, and both are present within gonadal tissues (52, 53). Generally, Dio1 and Dio2 are responsible for catalyzing the conversion of T4 into the more active T3, and Dio3 is responsible for the conversion of T3 and T4 into the inactive metabolites reverse T3 (rT3) and T2 respectively. Previous studies have demonstrated that the maintenance of a baseline level of active THs by deiodinases could be necessary for vertebrate testes development. In teleosts, *dio2* and *dio3* mRNA levels are higher in the testes than in the ovaries of the striped parrotfish *Scarus iseri* (73). In *O. mykiss*, testes are characterized by higher transcripts of *dio2*, and *dio2* expression, which is dependent on spermatogenic stages, increasing at the beginning of spermatogenesis (74). In the present study, no sexual dimorphic expression of *dio1*, *dio2*, *dio3a*, *tr*, and *tr* were found between testes and ovaries, Likewise, no changes in their expression levels were found after T4 or MET treatment by RNA-Seq. Yet, dio3 was identified as DEGs between C_XX and C_XY fugu. Both T4 and MET treatment downregulated its expression in XY fugu. These results suggest that T4 or MET treatment had an effect on thyroid hormone homeostasis. Previous studies have shown that the androgen axis directly regulates TH synthesis, and there is considerable crosstalk between the androgen and TH axes (52, 53). In teleost fish, the main endogenous androgen is 11-ketotestosterone (11-KT) (75), and the steroidogenic enzymes 11-hydroxylase (cyp11c1) and 11-hydroxysteroid dehydrogenase 2 (hsd11b2), are responsible for converting testosterone and androstenedione to 11-KT (76,78). Here, the expression of *cyp11c1* was suppressed in the T4 and MET-treated XY fugu. Taken together, these results suggested that T4 and MET treatment downregulated the expression of *dio3* through suppression the expression of *cyp11c1*. Further research on these potential regulatory and feedback mechanisms should be conducted. Since the amount of total RNA extracted from gonads is limited, qPCR only verified the DEGs in our study. Detailed expression profiles of *dio1*, *dio2*, *dio3a*, *dio3*, *tr*, and *tr *in the XX and XY gonads should be examined, and the functional analysis of those genes in the process of sex differentiation in fugu should be conducted in the future.

In conclusion, the present study provides the first evidence that MET treatment causes masculinization in teleost fish. The effects of MET-induced masculinization in *T. rubripes* may act primarily *via* suppression of the expression of *foxl2* and *cyp19a1a*, and stimulation of the expression of *dmrt1*. Moreover, effects of higher concentrations of T4, or different concentrations of T3, on sex differentiation should be tested in the near future.

## Data Availability Statement

The datasets presented in this study can be found in online repositories. The names of the repository/repositories and accession number(s) can be found below: https://www.ncbi.nlm.nih.gov/, SRR12364833 https://www.ncbi.nlm.nih.gov/, SRR12358231 https://www.ncbi.nlm.nih.gov/, SRR12364848 https://www.ncbi.nlm.nih.gov/, SRR12364847 https://www.ncbi.nlm.nih.gov/, SRR12364850 https://www.ncbi.nlm.nih.gov/,SRR12364849.

## Ethics Statement

The animal study was reviewed and approved by Care and Use of Laboratory Animals in Dalian Ocean University, Dalian, China and the animal study ethical committee of Dalian Ocean University and comply with Chinese laws. Written informed consent was obtained from the owners for the participation of their animals in this study.

## Author Contributions

Conceived and designed the experiments: HY, QL, and YL. Performed the experiments: ZY, XS, and JJ. Collected sample and performed the analyses: ZY, YW, XS, JJ, BL, LZ, and H Y. Wrote the paper: ZY, XS, and HY. All authors contributed to the article and approved the submitted version.

## Funding

This research was supported by National Key R&D Program of China (2019YFD0900503), Youth Program of National Natural Science Foundation of China (31902347), Projects for Dalian Youth Star of Science and Technology (2019R0), Key R&D Program of Liaoning Province (2019JH2/10200015), Scientific, Technological and Innovation Program of Dalian (2018J12SN069) and General Project of Education Department of Liaoning Province (JL201904).

## Conflict of Interest

The authors declare that the research was conducted in the absence of any commercial or financial relationships that could be construed as a potential conflict of interest.
